# An Incidental Myxoma Hidden in Chest Pain

**DOI:** 10.7759/cureus.45340

**Published:** 2023-09-16

**Authors:** Joana Gomes Cochicho, José Miguel Silva, Rita Louro, Isabel Lavadinho

**Affiliations:** 1 Internal Medicine Department, Hospital Doutor José Maria Grande, Portalegre, PRT; 2 Internal Medicine Department, Unidade Local de Saúde do Norte Alentejano, Portalegre, PRT

**Keywords:** echocardiogram, cardiac tumor, myxoma, obstructive symptoms, chest pain

## Abstract

Chest pain is a very common symptom in an emergency context. Its differential diagnosis is extensive and includes some conditions that require immediate recognition and intervention. It can also be a symptom associated with rarer diagnostic possibilities. Here, we report the case of a 53-year-old woman admitted to the emergency department due to chest pain and initially diagnosed with non-ST elevation acute myocardial infarction. A transthoracic echocardiogram revealed a large hyperechogenic round mass, suggestive of a left atrial cardiac myxoma. Coronary angiography showed no significant lesions. The patient underwent cardiac surgery with excision of the mass, whose histological diagnosis was atrial myxoma. The immediate postoperative period was uneventful, and the patient was discharged asymptomatic and without echocardiographic changes. Cardiac tumors are a rare finding, of which myxomas are the most common. Symptoms can typically result from embolism, obstruction, or constitutional symptoms. A myxoma presenting as acute chest pain and mimicking an acute coronary syndrome is an uncommon finding. This case reminds us of an extremely rare differential diagnosis of chest pain and awakens us to the usefulness and importance of using echocardiography as a diagnostic tool.

## Introduction

Internists in emergency departments frequently encounter chest pain as a symptom. A thorough characterization and careful examination are imperative for a proper diagnosis [1,2]. Its differential diagnosis includes a wide range of pathologies of varying severity. For this reason, it is crucial to rapidly identify life-threatening causes, such as aortic dissection, pulmonary embolism, tension pneumothorax, cardiac tamponade, or acute coronary syndrome [1-3].

Primary percutaneous angioplasty is recommended for certain cases of acute coronary syndrome, which should be performed as soon as possible. Hence, for this purpose, the approach to a patient with acute chest pain necessarily involves performing instantly an electrocardiogram, in order to identify the candidates for primary percutaneous angioplasty and direct them immediately to a cardiac catheterization laboratory [3,4].

The diagnosis of acute coronary syndromes without ST-segment elevation involves the measurement of markers of myocardial ischemia. In recent years, high-sensitivity troponin has been shown to be the most sensitive marker [1]. However, it presents a relatively lower specificity and could be elevated in several acute situations, including aortic valvular disease or hypertrophic cardiomyopathy [5].

## Case presentation

A 53-year-old female patient with a past medical history of arterial hypertension, osteoarthritis, and active smoking (3.5 smoking pack years). The patient took bisoprolol 1.25 mg, perindopril 5 mg + indapamide 1.25 mg, and acemetacin 90 mg as a daily medication. She was admitted to the emergency department due to retrosternal oppressive chest pain radiating to the left upper limb, with a duration of three to four hours. The pain started at rest and had no relieving or aggravating factors, and the patient denied other accompanying symptoms. The patient's physical examination showed hemodynamic stability, with normal blood pressure and heart rate. During cardiac auscultation, regular rhythmic sounds were observed, as well as a mild diastolic murmur in the apex. Pulmonary auscultation and the remaining physical examination showed no abnormalities.

**Figure 1:**
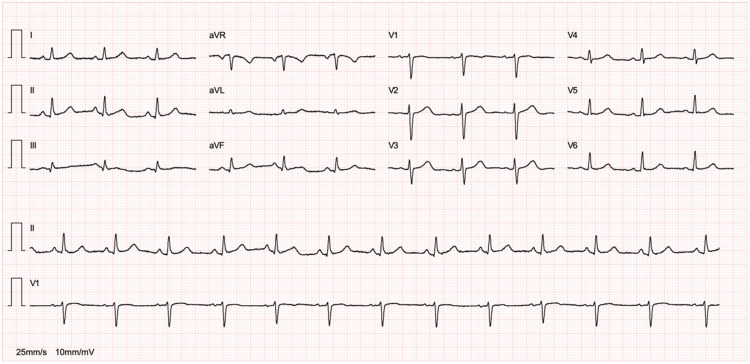
ECG on admission showing sinus rhythm, with no changes suggestive of acute ischemia

An electrocardiogram was performed, which did not show significant ST-segment elevation or other changes consistent with acute ischemia (Figure 1).

The only analytical alteration was a slight elevation in high-sensitivity troponin, with an upward momentum. High-sensitivity troponin at admission was 8.59 ng/L, after three hours was 39.66 ng/L, and after six hours was 189.70 ng/L. A working diagnosis of non-ST segment elevation myocardial infraction was established.

This hospital does not have a cardiac catheterization laboratory, which resulted in a delay of more than 24 hours in performing coronary angiography, and the patient received a loading dose of aspirin and ticagrelor. Analgesia was initiated, with a clear improvement in pain.

**Figure 2:**
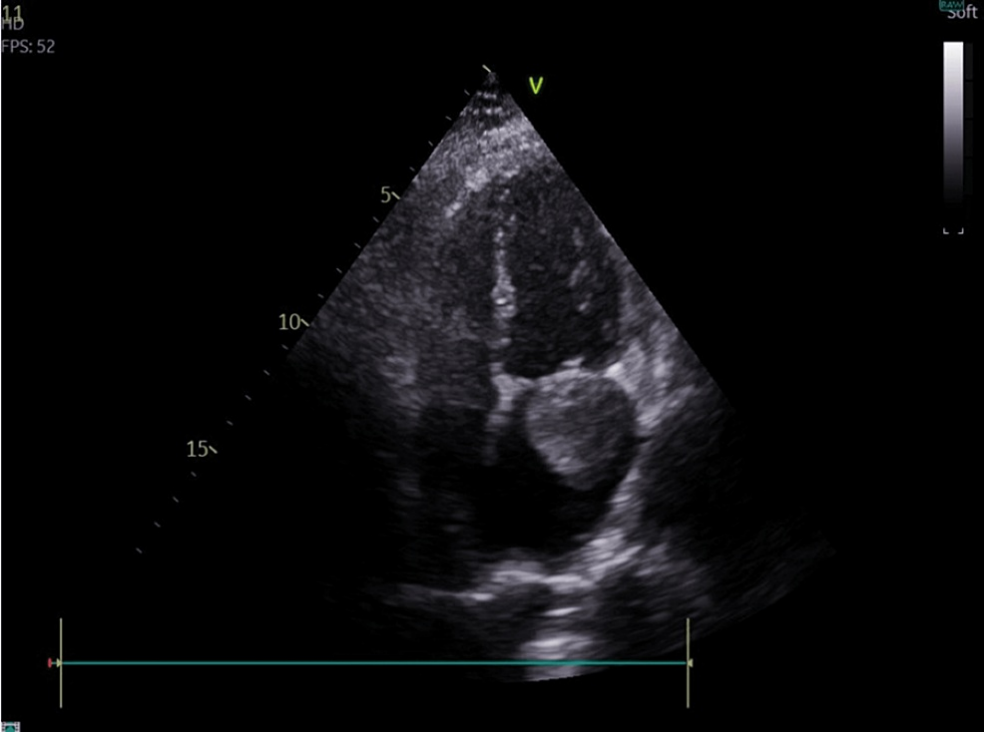
Echocardiographic image obtained in the four-chamber apical view, showing the myxoma in the left atrium

**Figure 3:**
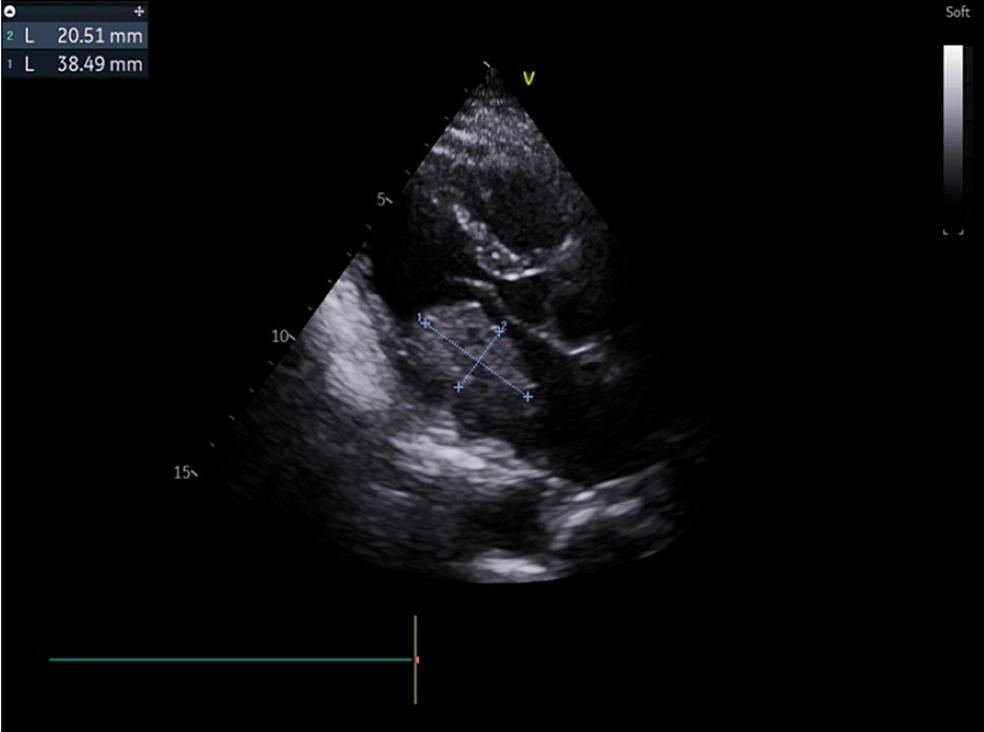
Echocardiographic image obtained in the parasternal long-axis view, showing the myxoma attached to the posterior leaflet of the mitral valve

**Figure 4:**
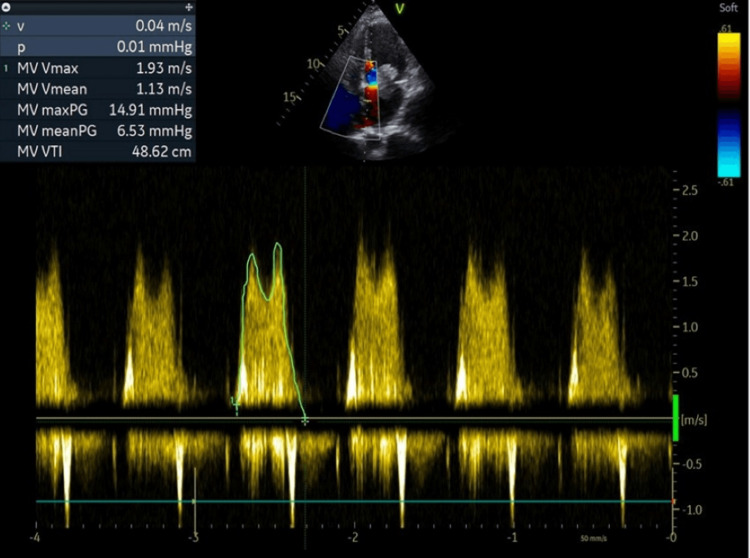
Echocardiographic image obtained in the four-chamber apical view, showing the mitral flow, compatible with moderate stenosis

Subsequently, a transthoracic echocardiogram was performed (Figures 2, 3, 4, and Video 1), showing normal LV function and no segmental wall motion abnormalities. However, the echocardiogram revealed the presence of a left atrial hyperechogenic rounded mass posterior to the mitral valve leaflet, measuring approximately 38 x 21 mm, causing moderate mitral stenosis.

**Video 1:** Echocardiographic video obtained in the four-chamber apical view, showing the myxoma in the left atrium

Considering the echocardiogram findings suggestive of myxoma, antiplatelet therapy was suspended. Furthermore, the patient was transferred to the cardiology department of the local area, where she completed the preoperative evaluation, including coronary angiography, which showed coronary arteries without significant lesions. Subsequently, she was transferred for cardiac surgery and underwent excision of the intracardiac mass. Histological evaluation of the operative specimen identified stellate and spindle cells with eosinophilic cytoplasm, arranged in aggregates and cords, around vessels and isolated, in a background of myxoid stroma. No mitoses or necrosis was documented, and the histological diagnosis was atrial myxoma.

The immediate postoperative period was uneventful, and the patient was discharged asymptomatic and without echocardiographic changes.

## Discussion

Primary cardiac tumors are extremely rare clinical entities. Approximately 50% to 75% of these masses correspond to myxomas, which are defined as non-neoplastic cardiac masses, resulting from an uncontrolled proliferation of mesenchymal cells. In the majority of cases, they are located in the left atrium [6-9]. From an epidemiological perspective, they primarily manifest in the sixth decade of life and are more common in women [6,7,9].

The clinical presentation of myxomas is variable and dependent on their size and location, which can include constitutional symptoms, resulting from embolization and/or obstruction. Typically, obstructive symptoms include shortness of breath, orthopnea, paroxysmal nocturnal dyspnea, palpitations, and hemoptysis and may also involve hepatic congestion, ascites, or syncope. Some of these symptoms may worsen in certain body positions [6,7,9].

In this case, the patient exhibited an atypical and sudden clinical manifestation, accompanied by an elevation in high-sensitivity troponin, which mimicked an acute coronary syndrome.

There are few reported cases of myxomas with this type of clinical presentation. In some of these cases, an elevation of myocardial necrosis markers was observed, resulting from embolization to the coronary arteries [8,10,11]. In a subgroup of these patients, as found in our clinical case, no coronary lesions were found by angiography. In a small percentage of cases, an ischemia with no obstructive coronary artery disease can be explained by coronary embolization. The absence of coronary artery lesions can be interpreted in the context of a high rate of recanalization [11-13].

However, taking into account the absence of segmental wall motion abnormalities, in the early echocardiogram performed on this patient, the chest pain could be consequent to the obstruction to the transmitral flow caused by the myxoma, identified on the echocardiogram, making this a rare case.

Obtaining an echocardiogram is crucial for the early diagnosis of rare causes, such as atrial myxoma, in atypical presentations of acute coronary syndrome.

In a specific case of myxomas, in addition to obtaining diagnostic confirmation and differentiating this type of mass from vegetations or thrombi, the echocardiogram allows for the identification of the location, size, site of attachment, and mobility. Echocardiography is an essential diagnostic tool for emergency physicians that cannot be overlooked. Its ability to characterize the mass aids in surgical preparation [9].

## Conclusions

Despite their benign clinical nature, myxomas can be associated with devastating complications. Considering that their presentation can be highly variable, depending on the location and characteristics of the mass, a high level of suspicion is important in the face of constitutional, embolic, and obstructive symptoms.

The echocardiogram is a crucial diagnostic tool that expedites referral for cardiac surgery. Furthermore, it plays a pivotal role in preoperative characterization, especially in cases requiring urgent surgery, as presented in this clinical case.
